# Synthesis, Characterization and Antibacterial Activity of Novel 1,3-Diethyl-1,3-bis(4-nitrophenyl)urea and Its Metal(II) Complexes

**DOI:** 10.3390/molecules22122125

**Published:** 2017-12-02

**Authors:** Hoda Pasdar, Bahare Hedayati Saghavaz, Naser Foroughifar, Mehran Davallo

**Affiliations:** Department of Chemistry, North Tehran Branch, Islamic Azad University, Tehran 1651153311, Iran; bahare.hedayati67@gmail.com (B.H.S.); nforoughifar@gmail.com (N.F.); m_davallo@yahoo.com (M.D.)

**Keywords:** dinuclear metal(II) complexes, monoclinic system, symmetrical urea, antibacterial activity, disc diffusion, broth dilution

## Abstract

A bioactive ligand and its dinuclear metal(II) complexes were synthesized and characterized by Fourier-transform infrared spectroscopy (FT-IR), ultraviolet-visible (UV-Visible), nuclear magnetic resonance (^1^H-NMR), mass spectroscopy and molar conductance measurements. The ligand has been crystalized in the monoclinic system with a P21/c space group. The biological activities of metal complexes were evaluated using disc diffusion and broth dilution methods. In vitro antibacterial activities of the ligand and their metal complexes were examined against two Gram-positive bacteria (*Bacillus subtilis* and *Staphylococcus aureus*) and two Gram-negative bacteria (*Escherichia coli* and *Serratia marcescens*) and compared to the standard drugs. It was found that metal complexes displayed much higher antibacterial activities and better inhibitory effects than that of the ligand and standard drugs. Among these complexes, the compound having Zn-metal showed greater antibacterial activity against all four tested bacteria and was more effective against *Serratia marcescens* with the zone inhibition diameter of 26 mm and MIC value of 31.25 µg/mL.

## 1. Introduction

In the past few decades, due to the changes in human habits and climate changes, bacterial infections have been a major cause of illness or death [1]. Antibiotics are essential to treat infections caused by bacteria. However, their overuse and misuse by humans have been linked to bacteria resistance, which is a severe public health problem [2]. To overcome this serious medical problem, the discovery of new types of antibiotics or the expansion of existing drugs is a very important and challenging issue [3]. Therefore, in recent years, research has been focused on developing new drugs, which may act through structural changes, to solve the problem of bacterial drug resistant [4].

**Figure 1:**
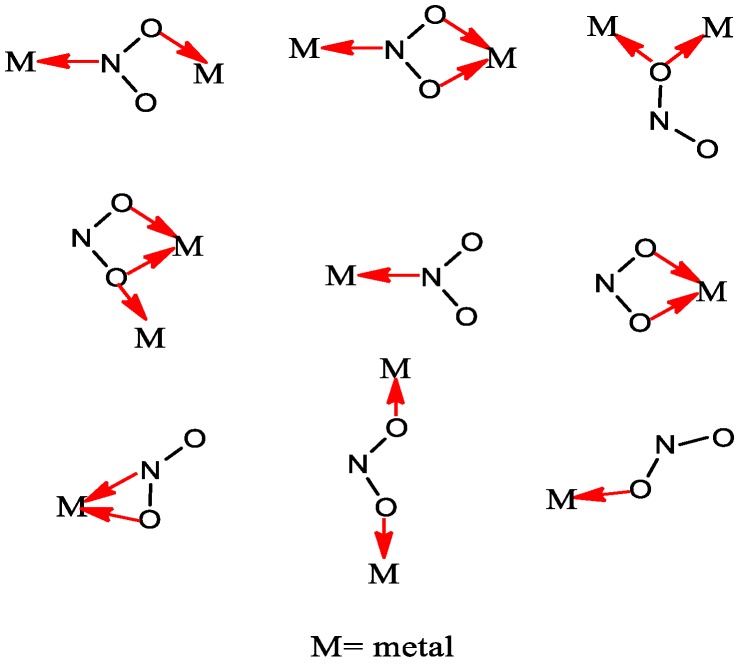
Different coordination modes of NO_2_ group.

Linkage isomerism is of major interest in the field of inorganic chemistry can be used to develop new compounds with potential pharmaceutical applications in the field of biomedicine [5,6,7]. Linkage isomerism occurs when an ambidentate ligand (e.g., NO_2_, SCN, CNO, etc.) binds to the metal center [8,9,10]. Nitro-nitrito metal complexes can be regarded as good examples of linkage isomerism, which was first described by Jørgensen and further developed through the syntheses of [(NH_3_)_5_CoNO_2_]Cl_2_ and [(NH_3_)_5_CoONO]]Cl_2_ complexes by Werner [11]. It must be noted that the NO_2_ group can be coordinated to the metal atom in different ways such as nitro (via the nitrogen), nitrito (via oxygen), chelating via both oxygens (nitrito-O,O′), and bridging nitro, usually via nitrogen and oxygen atoms. It has been shown that the coordination mode of nitrite ion depends on its nature and the stereochemical environment which surrounds the metal ion [12,13,14,15]. Apart from the wide range of NO_2_ binding modes in mononuclear metal complexes, their potential tendency to act as bidentate ligand must be mentioned [16]. Various coordination modes of NO_2_ group are shown in Figure 1.

Coordination chemistry of NO_2_ complexes is very attractive due to the significant role they play in different biosystems [17]. It appears that the biological function of nitrite ion (NO_2_) is contingent upon the nitrite reaction with metalloproteins which results in greater diversity of binding modes of the nitrite ligand and therefore can provide several reaction pathways that can lead to numerous biological species [18]. It has been reported that the presence of nitrite ion in mammalian biological systems at micromolar concentrations can play a crucial role by serving as a biological reserve of the bioregulatory agent for nitric oxide [19]. It has also been found that various metal complexes synthesized from the same ligand may display diverse biological properties [20]. The present study reports on the synthesis, characterization and antibacterial activities of the ligand and its metal complexes.

## 2. Results and Discussion

### 2.1. Characterization of Ligand

**Figure 2:**
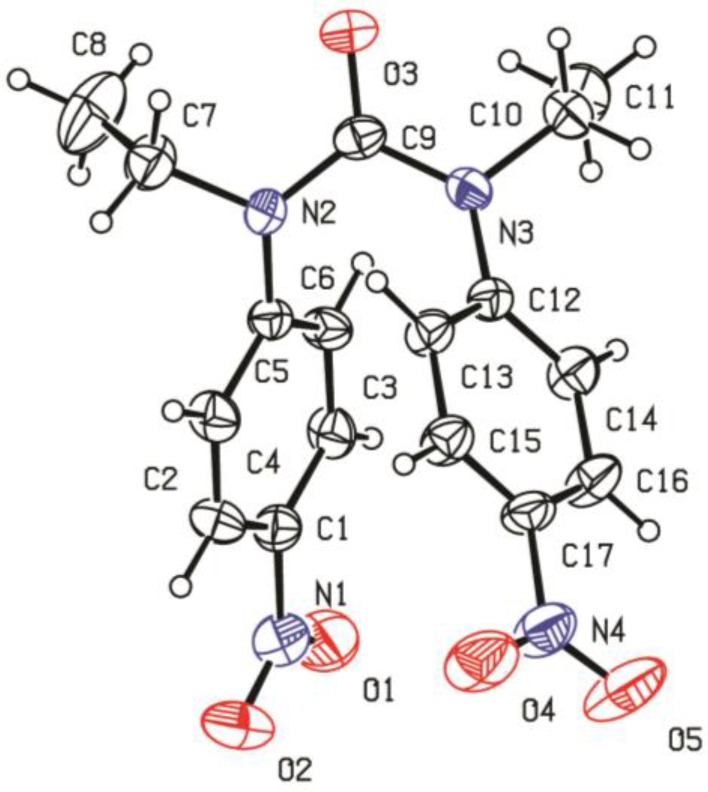
Crystal structure of ligand.

**Figure 3:**
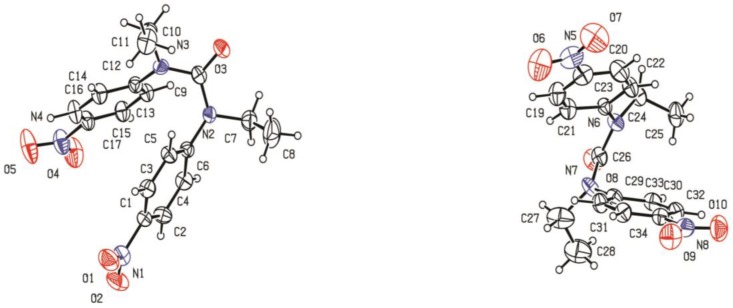
Unit cell of C_17_H_18_N_4_O_5_.

The symmetrical NO_2_-ligand (L) was synthesized by the reaction of n-Ethyl-4-nitro aniline and phosgene with a good yield (92%). A perspective view with the atom labeling of the ligand is given in Figure 2. In the X-ray crystallographic analysis, each unsymmetrical unit cell consists of two ligands (Figure 3).

Selected bond lengths and angles of the ligand (L) are given in Table 1.

In the ^1^H-NMR spectrum of the ligand, doublet peaks appear at 7.92 (J = 8 Hz) and 7.17 (J = 8 Hz) ppm, and are attributed to the aromatic protons. The CH_2_ and CH_3_ groups appear at 3.74 (J = 6.75 Hz) and 1.093 (J = 12 Hz) ppm, respectively, indicated the ethyl group of the ligand.

### 2.2. Characterization of Metal Complexes

Physical properties of the synthesized compounds are presented in Table 2. The NO_2_ group of ligand can bind to the metal atoms in a variety of different ways. As an ambidentate ligand, it can coordinate via nitrogen (nitro) or via oxygen (nitrito), and, as a bidentate ligand, it can coordinate via the two oxygens (nitrito-O,O′), and via one nitrogen and one oxygen or via a singlet oxygen atom. The synthetized metal complexes were obtained in moderate yield (50–83%), as shown in Table 2. In all experiments, ligand and metal salts were reacted (molar ratio 1:2) in ethanol solution under reflux condition. The prepared metal complexes were colored; stable; insoluble in methanol, ethanol, acetonitrile and chloroform; and well soluble in DMSO and DMF.

**Figure 4:**
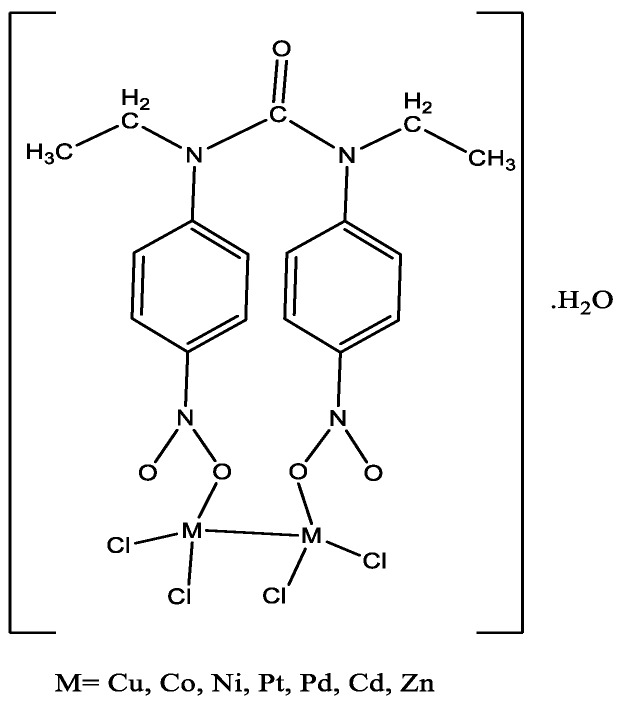
Proposed structure of metal complexes.

Metal complexes were characterized by Fourier-transform infrared spectroscopy (FT-IR), ultraviolet-visible (UV-Visible), and mass spectroscopy. The proposed structure of metal complexes is shown in Figure 4.

The molar conductivity of metal complexes was measured using the conductometric method. The molar conductivity of metal complexes was calculated using Equation (1):

(1) $$\Lambda_{m}=\frac{\kappa }{C}$$

where κ the measured conductivity and C is the concentration of the solutions. The molar conductance values of all the complexes were in the range 8–20 Ώ^−1^ cm^2^ mol^−1^ which indicated these complexes were non-electrolytes.

Important FTIR data are summarized in Table 3. As can be observed in Table 3, the C-H (aromatic) and C-H (aliphatic) bands for the ligand appear at 3109 cm^−1^ and 2975 cm^−1^, respectively. However, for the metal complexes, these bands were shifted to lower values in comparison to the parent ligand. The bands in the range of 1509–1591 cm^−1^ and 1309–1495 cm^−1^ correspond to the NO_2_ group of the ligand. The band observed at 1668 cm^−1^ is related to C=O stretching of the ligand. All complexes showed a characteristic band in the range of 510–600 cm^−1^ that is believed to be predominantly due to the metal–oxygen stretching. These results confirmed the nitrito isomer formation. The appearance of a broad peak in the range of 3442–3332 cm^−1^ indicated the presence of H_2_O in the structure of all the complexes.

The electronic absorption spectra of the ligand and its metal complexes were recorded in DMSO solution and the data obtained are given in Table 4. The ligand showed two peaks at 260 and 340 nm, which are attributed to the ᴨ→ ᴨ* and ᴨ→ ᴨ* transitions, respectively. For all complexes, except for Cu, Co and Ni, strong peaks of these transitions are present between 250 and 350 nm (for Cd complex; between 245 and 295 nm for Zn complex; between 270 and 350 nm for Pd complex; and between 260 and 350 nm for Pt complex). For Cu, Co and Ni complexes, an absorption band at 320, 350 and 295 nm is assigned to the ᴨ→ᴨ* transitions, respectively. For Zn and Cd complexes, having d^10^ electron configuration, d–d transitions were not observed for these complexes. The absorption bands at 490 nm for Cu, at 600 and 700 nm for Co, a very weak low-intensity at 510 nm for Pd, at 410 nm for Ni, and at 560 nm for Pt are associated with the d–d transitions of the metal atoms. 

**Figure 5:**
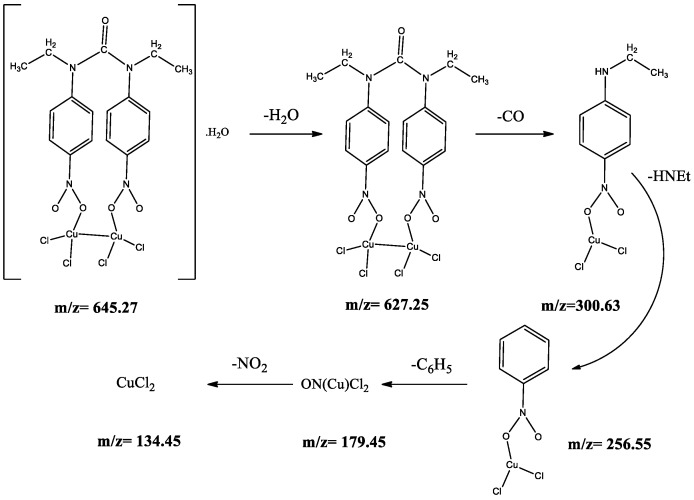
Mass fragmentation pattern of the CuL complex.

The Mass spectra of the synthesized compounds confirmed the proposed structures. The molecular ion peak for the Cu(II), Ni(II) Co(II), Pd(II), Cd(II) and Zn(II) complexes observed at *m*/*z* = 645, 636, 636, 730, 742 and 648, respectively, are equal to the molecular weight of these complexes. The other peaks in the mass spectrum are attributed to the fragmentation of complex inside the molecule. The fragmentation pattern of the Cu complex is shown in Figure 5.

### 2.3. Antibacterial Activity

**Figure 6:**
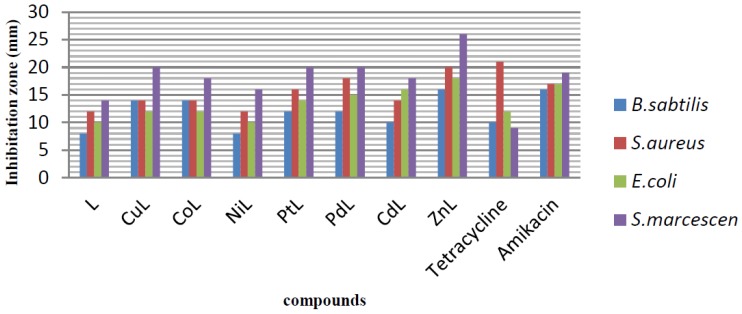
Graphical presentation of antibacterial activity as inhibition zone diameters (mm) of ligand (L) and its metal complexes against pathogenic strains based on disc diffusion method.

The in vitro antibacterial activities of the ligand, its metal complexes and standard antibiotic drugs were investigated using two Gram-positive bacteria (*Bacillus subtilis* and *Staphylococcus aureus*) and two Gram-negative bacteria (*Escherichia coli* and *Serratia marcescens*) by applying the disc diffusion and broth microdilution methods. The antibacterial activities of the ligand and all the complexes were compared to standard drugs (tetracycline and amikacin). The evaluation of antibacterial effect, conducted by measuring the diameter of growth inhibition, is shown in Figure 6, and the determination of minimum inhibitory concentration (MIC) is listed in Table 5. In general, the metal complexes displayed better inhibitory effect when compared to the ligand against all tested bacteria strains with zone inhibition in the range of 8–26 mm. Among all of the metal complexes, the compound with Zn transition metal showed highest antibacterial activities with the zone inhibition diameter ranging from 16 to 26 mm against both Gram-positive and Gram-negative bacteria strains, compared to the standard antibacterial drugs with the zone inhibition diameter ranging from 9 to 21 mm, as is evident in Figure 6. From the antibacterial results listed in Table 5, it can be seen that the ligand, its metal complexes and standard drugs (tetracycline and amikacin) showed different inhibition activities against various kinds of bacteria. Antibacterial activity of ligand against *Bacillus subtilis* showed less effectiveness with MIC value of 1000 µg/mL, whereas, against *Staphylococcus aureus*, *Escherichia coli*, and *Serratia marcescens* bacteria, showed better inhibition activity with similar MIC value of 500 µg/mL. Antibacterial activities of the seven kinds of complexes showed that majority of these complexes had effective activities against the bacteria strains. In general, the MIC of these complexes was better than that of the ligand and standard drugs. This phenomenon can be explained based on Tweedy’s chelation [21,22]. The chelation reduces the polarity of metal cation to some extent due to the overlap of the ligand orbital and partial sharing of the positive charge of the metal ion. The chelation increases the delocalization of p-electrons over the whole chelate ring and enhances the lipophilicity of the complexes which, in turn, increases the penetration of the complexes into lipid membranes, and results in blockage of metal sites in the enzymes of the microorganisms. In addition, metal complexes hinder the respiration process of the cell and, block the synthesis of proteins and prevent further growth of the organism. The highest inhibition activity against *Serratia marcescens* bacteria was observed for Zn-metal complex with MIC value of 31.25 µg/mL, followed by Pd-metal complex with MIC value of 62.5 µg/mL, which were 16-fold and 8-fold higher than the respective ligand. The MIC value for Zn-metal complex suggested that this compound had much stronger antibacterial activities (Table 5). It is evident from Table 5 that the changes in different metal complexes led to the changes of the activities against the used pathogenic strains. For metal complexes, the antibacterial activities against *Bacillus subtilis* increased in the order: Zn = Cd = Pd > Pt = Ni = Co = Cu. For metal complexes, the antibacterial activities against *Escherichia coli* increased in the order: Zn = Cd = Pd > Pt = Co = Cu > Ni. For metal complexes, the antibacterial activities against *Serratia marcescens* increased in the order: Zn > Pd > Cd = Pt = Co = Cu > Ni.

## 3. Materials and Methods

### 3.1. Materials

All solvents and reagents were obtained from Merck (Darmstadt, Germany) or Sigma Aldrich (St. Louis, MO, USA), and used without further purification. The completion of reactions monitored by TLC (thin layer chromatography) using n-hexane/EtOAC (in a ratio 1:5) as an eluent. Melting points of the compounds were measured by an electro-thermal melting point apparatus and were not corrected. Infrared spectra of the synthesized compounds were recorded with a Shimidzo 300 spectrometer in the region of 4000–400 cm^−1^ using Potassium bromide pellets. UV-visible spectra were obtained on a Cary 100 spectrophotometer in DMSO solutions. The Mass spectra were performed at 70 eV at 230 °C with Agilent technologies. The molar conductance of the complexes in DMSO (1 × 10^−3^ M solution) was carried out at 25 °C using Oakton EC Tester (Vernon Hills, IL, USA) 11 dual-range, conductivity tester. ^1^H-NMR spectra of NO_2_-ligand were recorded on a Brucker AMX 250 MHz spectrometer (Billerica, MA, USA) in DMSO-*d*_6_ solvent using tetramethylsilan as an internal reference.

### 3.2. Synthesis of the Ligand

The ligand was prepared according to the previous literature with slight modification [23]. In a 250 mL clean two-necked round-bottomed flask, containing an ice-cooled mixture of *N*-ethyl-4-nitroaniline (16.6 g, 0.1 mol) in dichloromethane (100 mL), a solution of phosgene in toluene (20%, 25 mL, 0.05 mol) was slowly introduced over a period of 30 min. The mixture was stirred in an ice bath for 3 h, and then left at room temperature overnight. After, it was dried under vacuum (2 mbar) at 60 °C, a solid residue was obtained. The obtained solid residue was then dissolved in dichloromethane (100 mL), and the resulting solution was washed with aq. HCl (100 mL), and with water (3 × 100 mL), respectively. The organic layer formed was separated and concentrated under vacuum (2 mbar) at 35 °C to give a thick oil product which was recrystallized via methanol, yielding a colorless product.

(16.4 g, 92%); M.p. 149–151 °C. Selected IR data (KBr, cm^−1^): 3109, 2975, 1668, 1591, 1373. ^1^H-NMR (250 MHz, DMSO-d_6_): 7.95 ppm (d, *J* = 8 Hz, 1, Ar-H), 7.17 ppm (d, *J* = 8 Hz, 1, Ar-H), 3.74 ppm (d, *J* = 6.75 Hz, 1, CH_2_), 1.09 ppm (t, *J* = 12Hz, 2, CH_3_). UV-vis (DMSO): λMax = 260, 340 nm. 

### 3.3. General Procedure for Preparation of Metal Complexes

A solution of (0.2 mmol) MCl_2_·xH_2_O in ethanol (25 mL) was prepared. Then, the solution of ligand (0.1 mmol) in ethanol (25 mL) was added to it and the resulting mixture was stirred for 1 h at ambient temperature and allowed to react under reflux for 3–5 h with stirring overnight. After the reaction was completed, the resulting solid was obtained from the solution by filtration. The resulting solid was washed and recrystallized via hot ethanol and finally dried in vacuum desiccator overnight. 

#### 3.3.1. Copper(II) Complex: [Cu_2_(L)Cl_4_]·H_2_O

Dark green solid; Yield: (75%); M.p. 240–242 °C. Molar conductance $\Lambda_{m}(\Omega^{-1}{\mathrm{cm}}^{2}{\mathrm{mol}}^{-1})$ in DMSO: 12. Selected IR data (KBr, cm^−1^): 3442, 2996, 2912, 1663, 1592, 1437, 1339, 699, 669, 510. UV-vis (DMSO): λ_Max_ = 320, 490 nm. Mass: [*m*/*z*]^+^ = 645.

#### 3.3.2. Nickel(II) Complex: [Ni_2_(L)Cl_4_]·H_2_O

Pale green solid; Yield: (70%); M.p. 230–232 °C. Molar conductance $\Lambda_{m}(\Omega^{-1}{\mathrm{cm}}^{2}{\mathrm{mol}}^{-1})$ in DMSO: 10. Selected IR data (KBr, cm^−1^): 3432, 2996, 2912, 1661, 1592, 1437, 1339, 701, 669, 600. UV-vis (DMSO): λ_Max_ = 295, 410 nm. Mass: [*m*/*z*]^+^ = 636.

#### 3.3.3. Cobalt(II) Complex: [Co_2_(L)Cl_4_]·H_2_O

Pale blue solid; Yield: (83%); M.p. 242–244 °C. Molar conductance $\Lambda_{m}(\Omega^{-1}{\mathrm{cm}}^{2}{\mathrm{mol}}^{-1})$ in DMSO: 8. Selected IR data (KBr, cm^−1^): 3439, 2996, 2912, 1661, 1515, 1437, 1311, 700, 669, 600. UV-vis (DMSO): λ_Max_ = 3500, 600, 700 nm. Mass: [*m*/*z*]^+^ = 636.

#### 3.3.4. Palladium(II) Complex: [Pd_2_(L)Cl_4_]·H_2_O

Black solid; Yield: (75%); M.p. > 300 °C. Molar conductance $\Lambda_{m}(\Omega^{-1}{\mathrm{cm}}^{2}{\mathrm{mol}}^{-1})$ in DMSO: 12. Selected IR data (KBr, cm^−1^): 3438, 2996, 2912, 1661, 1515, 1437, 1339, 700, 669, 600. UV-vis (DMSO): λ_Max_ = 260, 350, 510 nm. Mass: [*m*/*z*]^+^ = 730.

#### 3.3.5. Platinum(II) Complex: [Pt_2_(L)Cl_4_]·H_2_O

Dark brown solid; Yield: (50%); M.p. > 300 °C. Molar conductance $\Lambda_{m}(\Omega^{-1}{\mathrm{cm}}^{2}{\mathrm{mol}}^{-1})$ in DMSO: 14. Selected IR data (KBr, cm^−1^): 3434, 2998, 2913, 1657, 1437, 1384, 702, 699, 550. UV-vis (DMSO): λ_Max_ = 280, 350, 560 nm. Mass: [*m*/*z*]^+^ = 910.

#### 3.3.6. Zinc(II) Complex: [Zn_2_(L)Cl_4_]·H_2_O

White solid; Yield: (80%); M.p. 250–252 °C. Molar conductance $\Lambda_{m}(\Omega^{-1}{\mathrm{cm}}^{2}{\mathrm{mol}}^{-1})$ in DMSO: 14. Selected IR data (KBr, cm^−1^): 3435, 2996, 2912, 1661, 1437, 1312, 701, 670, 510. UV-vis (DMSO): λ_Max_ = 270, 350 nm. Mass: [*m*/*z*]^+^ = 648.

#### 3.3.7. Cadmium(II) Complex: [Cd_2_(L)Cl_4_]·H_2_O

Milky solid; Yield: (78%); M.p. 282–284 °C. Molar conductance $\Lambda_{m}(\Omega^{-1}{\mathrm{cm}}^{2}{\mathrm{mol}}^{-1})$ in DMSO: 20 IR (KBr, cm^−1^): 3435, 2996, 2912, 1661, 1437, 1311, 699, 669, 550. UV-vis (DMSO): λ_Max_ = 245, 295 nm. Mass: [*m*/*z*]^+^ = 742.

### 3.4. In Vitro Antibacterial Activity

Antibacterial activities of all synthesized compounds were evaluated against pathogenic strains by applying agar disc diffusion and broth dilution methods [24,25]. The tests were performed using the methodology described in the guidelines of the Clinical and Laboratory Standards Institute (CLSI). The bacterial pathogens used in this study were *Bacillus subtilis* (ATCC: 6633) and *Staphylococcus aureus* (ATCC: 6838) as Gram-positive bacteria; and *Escherichia coli* (ATCC: 25922) and *Serratia marcescens* (ATCC: 13880) as Gram-negative bacteria. Each of the bacterial strains were cultured onto Muller–Hinton agar (MHA) plate and incubated for 18–24 h at 35 °C. The turbidity of all microorganisms was adjusted to 0.5 McFarland turbidity standards to obtain a 1.5 × 10^8^ CFU/mL suspension.

#### 3.4.1. Disc Diffusion Method

All the examined compounds were prepared by dissolving 20 mg of each compound in 1 mL of DMSO. Thus, DMSO was used as a negative control for all the samples examined. Tetracycline and Amikacin were used as standard drugs. A bacterial culture (which has been adjusted to 0.5 McFarland) was used to lawn Hinton agar plates using a sterile swab. Paper discs of 8 mm diameter were impregnated individually with a constant amount (100 μg/mL) of the compounds. Plates were incubated at 37 °C for 18–24 h and the antibacterial activity of each test sample was determined by measuring the diameter of zone of inhibition and comparing with standard drug. The antibacterial behavior of each test sample was repeated twice. No inhibition zone was found for DMSO sample. 

#### 3.4.2. Determination of Minimal Inhibitory Concentration (MIC)

Antibacterial activities of all the compounds and standard drugs were also evaluated using the microdilution method. Each test compound and standard drug individually was prepared in DMSO to obtain 2000 µg/mL concentration (stock solution). The aim of the broth micro-dilution method was the evaluation of the lowest concentration of the examined antibacterial agent to inhibit the visible growth of the microorganism being investigated. Muller–Hinton Broths (MHB) was used as bacterial nutrients. The inoculum size of all strains was adjusted to 1.5 × 10^8^ CFU /mL using 0.5 McFarland standard solution for each antibacterial compound and standard drug (tetracycline and amikacin). Thirty-nine tubes of 5 mL volume were used in 3 rows such that each row contained 13 tubes. Afterwards, 1 mL of Muller–Hinton Broth (for Row 1 and 2) and 1 mL of standard drug broth (for row 3) were added in Tubes 1–13 in each row. Then, 1 mL of the antibacterial compound (stock solution) was added to the first tube in each row and mixed. After mixing, 1 mL of the first tube in each row was serially carried over to the second tube in the same row, mixed and the content of the second tube was transferred to the third tube in each row. This serial dilution was repeated to Tube 12 in each of the rows and 1 mL of Tube 12 was discarded. Tube 12 had no bacteria and was used as a negative control. Tube 13, without antibacterial agent, was used as a positive control. Thus, the micro-dilution provided antibacterial concentrations of 1000, 500, 250, 125, 62.5, 31.25, 15.62, 7.81, 3.90, 1.95, 0.975, and 0.487 µg/mL, respectively. Finally, 100 µL of bacteria suspension was added to Tubes 1–11 and 13 in Rows 1–3 and were incubated for 24 h at 37 °C. The highest dilution of active sample to inhibit evident growth of the microorganism was expressed as the MIC.

### 3.5. X-Ray Data Collection and Refinement of Crystal Structure of Ligand

Crystals of 1,3-diethyl-1,3-bis(4-nitrophenyl)urea were grown by slow evaporation from methanol solution. The X-ray diffraction measurement was carried out on STOE IPDS- 2/2T diffractometer with monochromated Mo Kα (λ = 0.71073 Å) irradiation. The crystal structure of the ligand obtained after one week, was characterized by X-ray crystallography (CCDC code: khc1252h contains the supplementary crystallographic data). The crystal data and structure refinement of the ligand are listed in Table 6. The refinement was carried out using SHELXL. The structure was analysed by direct method. These data can be observed from the Cambridge Crystallographic Data Center via www.ccdc.com.ac.uk/data-request/cif.

## 4. Conclusions

We successfully synthesized novel Cu(II), Ni(II), Co(II), Zn(II), Cd(II), Pd(II) and Pt(II) complexes derived from 1,3-diethyl-1,3-bis(4-nitrophenyl)urea. The structure of the parent ligand was investigated by FTIR, ^1^H-NMR and crystallographic spectroscopy. The corresponding metal complexes were characterized using FTIR, UV-Vis and Mass spectroscopy. The FTIR data showed that the ligand bonds to the metal ions via oxygen atom and these data confirmed the formation of nitrito isomer. The measured molar conductivity of the metal complexes was found to be in the range of 8–20 Ώ^−1^ cm^2^ mol^−1^ which indicated these complexes were non-electrolytes. By comparing the ligand and its metal complexes, it was found that, in general, metal complexes showed better inhibitory effect and also exhibited much lower MIC values against all the tested bacteria strains. Among these metal complexes, compound with Zn metal transition showed highest antibacterial activities against both Gram-positive and Gram-negative bacteria strains, when compared to the standard antibacterial drugs. The highest inhibition activity was observed for Zn metal complex against *Serratia marcescens* bacteria. This suggested that Zn compound would be better therapeutic drug for antibacterial treatment. 

## Figures and Tables

**Table 1:** Selected bond lengths (**Å**) and bond angles (°).

Bond (Å)		Angle (°)	
O(1)-N(1)	1.223(4)	O(1)-N(1)-O(2)	123.7(4)
O(2)-N(1)	1.223(4)	O(1)-N(1)-C(1)	118.2(4)
O(3)-C(9)	1.222(4)	O(2)-N(1)-C(1)	118.0(4)
O(4)-N(4)	1.204(4)	C(9)-N(2)-C(6)	122.8(3)
O(5)-N(4)	1.214(4)	C(9)-N(2)-C(7)	116.6(3)
O(6)-N(5)	1.213(4)	C(6)-N(2)-C(7)	118.6(3)
O(7)-N(5)	1.207(4)	C(9)-N(3)-C(12)	123.4(3)
O(8)-C(26)	1.213(4)	C(9)-N(3)-C(10)	116.0(3)
O(9)-N(8)	1.222(4)	C(12)-N(3)-C(10)	119.5(3)
O(10)-N(8)	1.221(4)	O(4)-N(4)-O(5)	123.0(4)
N(1)-C(1)	1.469(5)	O(4)-N(4)-C(17)	118.7(4)
N(2)-C(9)	1.383(4)	O(5)-N(4)-C(17)	118.3(4)
N(2)-C(6)	1.413(4)	O(7)-N(5)-O(6)	125.4(5)
N(2)-C(7)	1.470(4)	O(7)-N(5)-C(18)	117.1(5)

**Table 2:** Physical properties of Ligand (L) and its metal complexes.

Compounds	M.W. (g/mol)	Yield (%)	Color	Conductivity Ώ cm^2^ mol^−1^	M.P. (°C)
L	358	92	Colorless	-	149–151
CuL	645	87	Dark green	12	149–151
CoL	636	83	Pale blue	8	242–244
NiL	636	70	Pale green	10	240–242
PtL	910	50	Dark brown	14	>300
PdL	730	75	Black	12	>300
CdL	743	78	Cream	20	282–284
ZnL	648	80	White	14	250–253

**Table 3:** The Fourier-transform infrared (FTIR) spectroscopy data of ligand (L) and its metal complexes.

Compounds	(C-H)aromatic	(C-H)aliphatic	(NO_2_)	(NO_2_)	(C=O)	(OH)water	(M-O)
L	3109	2975	1533	1373	1668	-	-
CuL	2996	2912	1592	1339	1663	3442	510
CoL	2996	2912	1592	1311	1661	3439	600
NiL	2996	2912	1592	1339	1661	3432	600
PtL	2996	2913	1437	1384	1657	3434	550
PdL	2996	2912	1592	1339	1661	3438	600
CdL	2996	2912	137	1311	1661	3435	550
ZnL	2996	2912	1437	1312	1661	3435	510

**Table 4:** Electronic spectra data of ligand (L) and its metal complexes.

Compounds	λ_Max_ (nm)	d-d Transitions
L	260, 340	-
CuL	320, 490	2B_1_→2A_1_
CoL	350, 600, 700	4A_1_→4B_1_ 4A_1_→4B_2_
NiL	295,410	3B_1_→3A_2_
PtL	280, 350, 560	3B_1_→3A_1_
PdL	260, 350, 510	3B_1_→3A_1_
CdL	295	-
ZnL	270, 350	-

**Table 5:** Minimal inhibitory concentrations (µg/mL) of ligand (L) and their metal complexes against pathogenic strains based on broth micro-dilution method.

Compounds	G(+)		G(-)	
	*B. subtilis*	*S. aureus*	*E. coli*	*S. marcescens*
L	1000	500	500	500
CuL	250	250	250	125
CoL	250	250	250	125
NiL	500	250	500	250
PtL	250	250	250	125
PdL	125	125	125	62.5
CdL	250	125	125	125
ZnL	125	125	125	31.25
Tetracycline	500	250	250	125
Amikacin	500	500	250	250

G(+) denotes Gram positive bacteria and G(−) denotes Gram negative bacteria.

**Table 6:** Crystal structure data of ligand.

Formula	C_17_H_18_N_4_O_5_
Formula weight	358.35
System	Monoclinic
Color/shape	Colorless/plate
Space group	P 21/c
a (A°)	15.516(3)
b (A°)	16.826(3)
c (A°)	14.975(3)
α (°)	90
β (°)	-
γ (°)	90
T (k)	298(2)
V (Å-3)	3645.2(14)
Z	8
Dcal (Mg/m^3^)	1.306
Absorption coefficient (mm^−1^)	0.098
Crystal size (mm)	0.5 × 0.4 × 0.25
θ Range (°)	2.42 to 25.00
Reflections collected	16170
Goodness-of-fit on F2	0.893
Data/restraints/parameters	6398/0/474
Final R indices	R1 = 0.0677, wR2 = 0.0935
R indices (all data)	R1 = 0.1928, wR2 = 0.1177
Largest diff. peak and hole (e/Å-3)	0.332 and −0.213

## Supplementary Materials

CCDS khc1252h contains the supplementary crystallographic data for Ligand, and can be obtained free of charge via www.ccdc.com.ac.uk/data-request/cif, or from the Cambridge Crystallographic Data Centre.
