# Supplementary material for: Synthesis, Characterization and Antibacterial Activity of Novel 1,3-Diethyl-1,3-bis(4-nitrophenyl)urea and Its Metal(II) Complexes
Source: Molecules. 2017 Dec 2;22(12):2125. doi: 10.3390/molecules22122125 (PMC6149768; doi:10.3390/molecules22122125)
Supplement: Supplementary file 1 [file molecules-22-02125-s001.zip › molecules-241809-supplementary/FTIR/FTIR.zip!/FTIR/Ni.pdf]

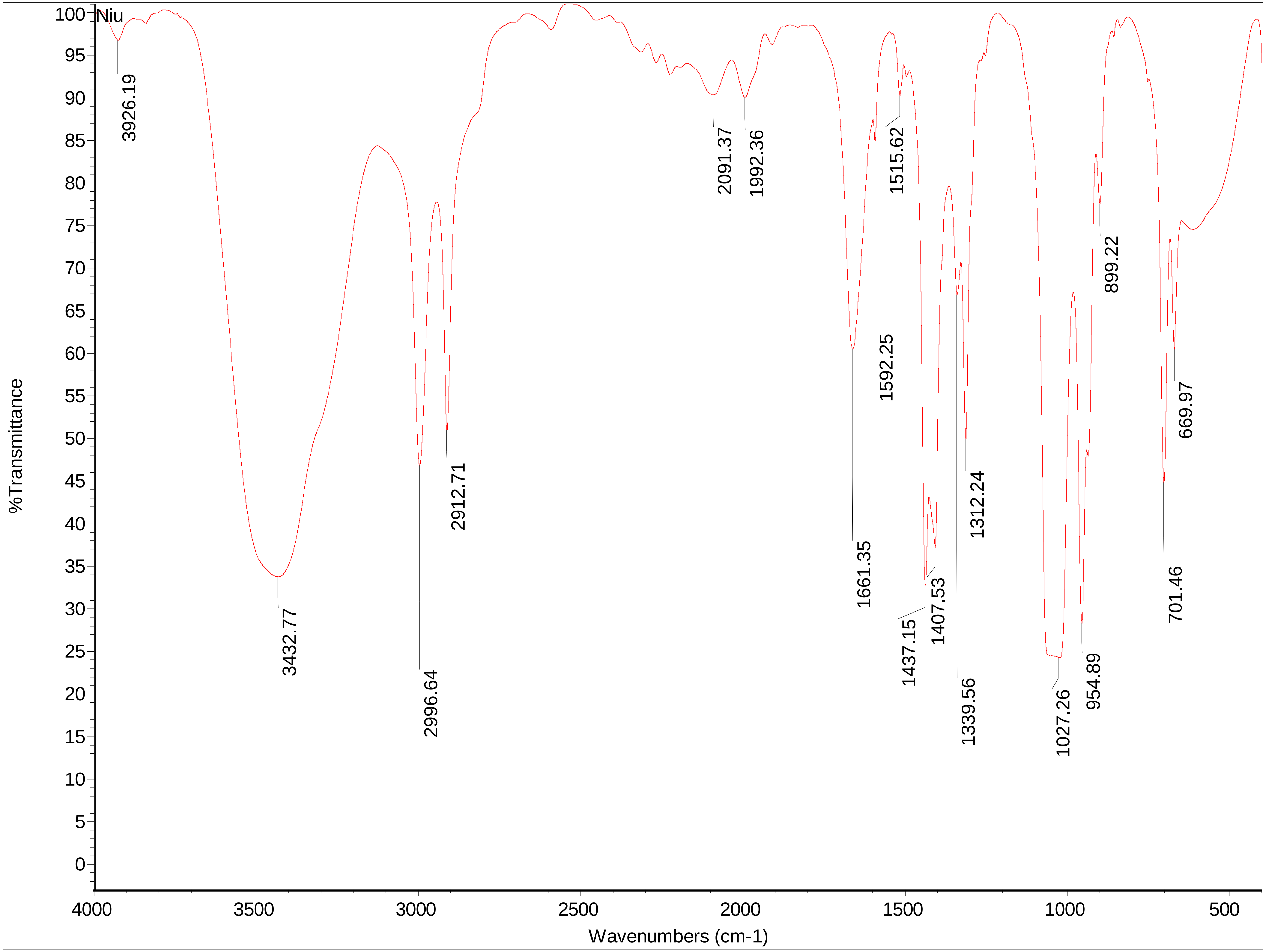


PDF created with pdfFactory Pro trial version www.pdffactory.com
